# High-Intensity Interval Training Attenuates Hepatic Fibrosis by Remodeling Lactate Metabolism in MASLD

**DOI:** 10.3390/metabo16060413

**Published:** 2026-06-13

**Authors:** Xuefei Chen, Jie Su, Wenhua Huang, Yanjun Li, Jing Zhang

**Affiliations:** 1 School of Kinesiology and Physical Education, Zhengzhou University, Zhengzhou 450000, China; chenxuefei@zzu.edu.cn; 2 Collage of P.E. and Sports, Beijing Normal University, Beijing 100875, China; 3 College of Physical Education and Health, Guangxi Normal University, Guilin 541006, China

**Keywords:** lactate metabolism, high-intensity interval training, metabolic dysfunction-associated steatotic liver disease, high-fat diet, hepatic fibrosis

## Abstract

**Highlights:**

**What are the main findings?**

• Chronic HIIT intervention significantly alleviates hepatic fibrosis and suppresses hepatic stellate cell activation in MASLD mice
• HIIT remodels the intrahepatic lactate metabolic axis by inhibiting LDHA-mediated production while concurrently enhancing MCT1-dependent efflux and gluconeogenic shunting via PC and PEPCK.

**What are the implications of the main findings?**

• These findings identify intrahepatic lactate homeostasis as a pivotal metabolic node tightly coupled with fibrogenesis, revealing an intricate molecular association between localized metabolic dysregulation and structural liver damage in MASLD.
• The study highlights the lactate metabolic axis as a potent non-pharmacological therapeutic target, providing a novel mechanistic rationale for utilizing HIIT as an evidence-based lifestyle intervention to manage MASLD.

**Abstract:**

**Background**: Metabolic dysfunction-associated steatotic liver disease (MASLD) has emerged as a global metabolic disorder. As a non-pharmacological intervention, the effects of high-intensity interval training (HIIT) on MASLD and its molecular mechanisms remain poorly understood. This study aimed to investigate whether HIIT could ameliorate high-fat diet (HFD)-induced liver fibrosis by recalibrating the intrahepatic lactate metabolic axis. **Methods:** An HFD-induced murine MASLD model combined with HIIT intervention was utilized to evaluate the therapeutic efficacy and underlying mechanisms. Hepatosomatic indices, histological architecture and fibrosis severity were examined. Lactate concentrations within the systemic circulation and hepatic parenchyma, alongside comprehensive lipid profiles, were measured. The expressions of genes and proteins involved in hepatic lactate metabolism were delineated via qPCR and Western blotting. **Results:** The 8-week HIIT intervention effectively improved liver lipid accumulation, hepatocellular injury, and oxidative stress caused by a high-fat diet. Fibrotic expansion and suppressed hepatic stellate cell activation were restricted markedly, as evidenced by the downregulation of collagen type I alpha 1 chain and alpha-smooth muscle actin(α-SMA). HIIT reversed the HFD-induced accumulation of lactate in both systemic circulation and liver tissues, which was found to positively correlate with hepatic α-SMA. Mechanistically, HIIT regulated the expression of the lactate metabolism-related proteins lactate dehydrogenase A and monocarboxylate transporter 1, while selectively enhancing the expression of the gluconeogenic enzymes. **Conclusions:** Our findings indicate that HIIT effectively ameliorated MASLD and associated hepatic fibrosis by remodeling the hepatic lactate metabolic axis, specifically through the suppression of lactate production and the enhancement of its clearance. These results indicate that targeting lactate homeostasis might be a promising therapeutic strategy for MASLD.

## 1. Introduction

Metabolic dysfunction-associated steatotic liver disease (MASLD) has rapidly evolved into an escalating public health crisis of global proportions. Unlike the traditional exclusionary criteria for non-alcoholic fatty liver disease, the current consensus defines MASLD through evidence of hepatic steatosis concomitant with at least one major metabolic dysregulation: overweight or obesity, type 2 diabetes, or a cluster of systemic metabolic risk abnormalities [1]. This chronic lipotoxic insult propagates a pathogenic cascade, driving hepatocyte injury, inflammation, hepatic fibrosis, and ultimately, hepatocellular carcinoma [2]. Despite its high prevalence of approximately 25–32% globally and severe clinical outcomes, there are currently no specific pharmacological treatments for MASLD, making the optimization of non-pharmacological interventions imperative [3,4].

In the absence of approved pharmacotherapies, lifestyle interventions, such as d regular exercise and dietary control, serve as the cornerstones of MASLD management [5,6]. Among diverse exercise modalities, high-intensity interval training (HIIT) has gained popularity for its time-efficiency and potent physiological adaptations. HIIT involves repeated bouts of relatively high-intensity exercise (≥85% of maximal oxygen uptake), with intervals of low-intensity exercise or short rest periods [7]. Compared with traditional continuous aerobic and resistance training, HIIT shows similar or better metabolic benefits in a much shorter time. Specifically, it provides robust advantages for MASLD patients by significantly improving cardiovascular function, enhancing peripheral and hepatic insulin sensitivity, and regulating lipid and glucose metabolism [8,9]. However, its specific therapeutic effects and underlying mechanisms in halting or reversing MASLD-associated liver fibrosis remain largely unexplored.

Increasing evidence indicates that lactate, a byproduct of anaerobic glycolytic metabolism, acts as a critical signaling molecule in liver diseases [10]. In the context of hepatic steatosis, impaired glucose utilization leads to reduced glucose oxidation and a shift toward anaerobic glycolysis, which further exacerbates insulin resistance. Metabolic imbalance caused intrahepatic lactate accumulation, which in turn promotes fatty acid synthesis and exacerbate hepatic steatosis. This led to the pathological progression from MASLD to metabolic dysfunction-associated steatohepatitis [11]. Furthermore, lactate can directly influence disease progression via lactylation. Research has demonstrated that histone lactylation is significantly upregulated in the livers of patients with MASLD [12], where it modulates genes associated with lipogenesis, inflammatory responses, and fibrosis [13]. This discovery highlights the lactate metabolism pathway as a promising therapeutic target for MASLD.

Although HIIT acutely increases systemic lactate, its long-term impact on the pre-existing lactate burden in liver fibrosis remains unclear. Given that lactate dysregulation functions as a critical driver of MASLD progression, it is essential to determine whether chronic HIIT exacerbates or alleviates this metabolic stress. Therefore, this study aimed to investigate the therapeutic effects of HIIT on MASLD-associated liver fibrosis using an HFD-induced murine model. We found HIIT intervention can alleviate the symptoms of MASLD and hepatic fibrosis by remodeling the hepatic lactate metabolic axis.

## 2. Materials and Methods

**Animals**

Six-week-old male C57BL/6J mice were sourced from the Beijing Vital River Laboratory Animal Technology Co., Ltd. (Beijing, China). The animals were housed in pairs within a specific pathogen-free (SPF) facility, maintained at a constant temperature (22 ± 2 °C) under a 12/12 h light-dark cycle. The animals were afforded ad libitum access to water and standard laboratory chow. All mouse groups underwent training and testing at 4:00 p.m. All animal care and experimental protocols complied with the guidelines of the Ethics and Animal Welfare Committee of Beijing Normal University (Ethical approval no. CLS-AWEC-B-2024-001).

**MASLD Modeling and Dietary Intervention**

A total of 28 mice were randomly assigned to three groups: a normal-diet sedentary group (CON, *n* = 8), a high-fat-diet sedentary group (HFD, *n* = 10), and a high-fat-diet-with-HIIT-treatment group (HFD + HIIT, *n* = 10). To establish the MASLD model, mice in the HFD and HFD + HIIT were maintained on a continuous high-fat diet (consisting of 60% fat, 20% carbohydrate, and 20% protein by caloric content) for a total duration of 16 weeks.

**Exercise Protocol**

During the final 8 weeks of the dietary intervention, the HFD + HIIT group engaged in an 8-week treadmill running regimen (5 days per week). After a 3-day acclimation period (15 min/session at 10–15 m/min), exercise capacity was evaluated via a maximum running speed (Vmax) test. The protocol began at 8 m/min, with the speed increasing by 1 m/min every 2 min until exhaustion (defined as remaining on the shock grid for >10 s without resuming running). This test was conducted every two weeks to recalibrate exercise intensity. Based on a previously established HIIT protocol [14], each HIIT session started with a 5 min treadmill run at 40% Vmax, followed by nine intervals of high-intensity running (1.5 min at 85% Vmax) interspersed with recovery (2 min at 45% Vmax), and ended with another 5 min run at 40% Vmax. To control environmental stress, mice in the CON and HFD groups were placed on a stationary treadmill for an equivalent duration.

**Sample Collection and Biochemical Analysis**

Twenty-four hours following the final exercise session, all mice were subjected to an overnight fast with unrestricted access to water and were then anesthetized via the intraperitoneal injection of pentobarbital sodium (100 mg/kg, Sigma-Aldrich, St. Louis, MO, USA). Blood samples were collected from the hearts of mice and the serum was isolated by centrifugation at 3000 rpm for 10 min, followed by storage at –80 °C. The liver and specific adipose depots—including inguinal white adipose tissue (iWAT), epididymal white adipose tissue (eWAT), and brown adipose tissue (BAT)—were excised, weighed, and immediately snap-frozen in liquid nitrogen for storage at –80 °C.

Biochemical parameters, including triglycerides (TG, A110-1-1), total cholesterol (CHO, A111-1-1), high-density lipoprotein cholesterol (HDL-c, A112-1-1), low-density lipoprotein cholesterol (LDL-c, A113-1-1), alanine aminotransferase (ALT, C010-2-1) and aspartate aminotransferase (AST, C010-2-1), malondialdehyde (MDA, A003-1-2) were quantified using assay kits from Nanjing Jiancheng Bioengineering Institute (Nanjing, China).

**Histological and Immunohistochemical Analysis**

After harvest, liver tissues were immediately fixed in 10% neutral-buffered formalin for 24 h. Subsequently, some tissues were dehydrated in a graded ethanol series, embedded in paraffin, and sectioned into 5-μm coronal slices. To evaluate hepatic neutral lipid accumulation, a portion was embedded in Tissue-Tek OCT (Sakura Finetek, Torrance, CA, USA) and sectioned into 5 μm coronal slices for Oil Red O staining (Solarbio, Beijing, China).

For immunohistochemical staining, the paraffin-embedded sections were deparaffinized, rehydrated, and subjected to antigen retrieval. To attenuate non-specific binding, sections were pre-blocked with 5% bovine serum albumin and subsequently incubated with alpha-smooth muscle actin (α-SMA) at 4 °C overnight. Following thorough washing, the sections were incubated with an HRP-conjugated secondary antibody. 3,3′-diaminobenzidine tetrahydrochloride (Servicebio, Wuhan, China) was used to visualize immunoreactive sites, followed by hematoxylin counterstaining to define the nuclei. Images were acquired using a ZEISS ImagerM1 microscope (Carl Zeiss Jena GmbH, Jena, Germany). Immunohistochemical quantification of the positive staining area was performed using ImageJ software (Version 1.53a). Results were expressed as the percentage of positive staining area.

**Western Blotting**

Total protein was harvested from snap-frozen liver tissues utilizing a standard lysis buffer enriched with a protease inhibitor cocktail. The BCA assay was used to determine protein concentrations. Equal amounts of protein samples were separated on 10–12% Bis-Tris gels and electrotransferred onto polyvinylidene difluoride (PVDF) membranes. The membranes were blocked with 5% non-fat milk for 1 h at room temperature, before being probed with primary antibodies against monocarboxylate transporter 1(MCT1, 20139-1-AP), lactate dehydrogenase B(LDHB, 14824-1-AP), lactate dehydrogenase A(LDHA, 19987-1-AP), α-SMA (14395-1-AP), and glyceraldehyde-3-phosphate dehydrogenase(GAPDH, 60004-1-Ig) (all from Proteintech, WuhanChina) at 4 °C overnight. Following TBST washes, the membranes were incubated for 1 h with an HRP-conjugated goat anti-rabbit IgG secondary antibody (1:8000, EarthOx, San Francisco, CA, USA). Protein bands were visualized using an enhanced chemiluminescence reagent (Millipore, Burlington, MA, USA) and captured with an Amersham Imager 680 (General Electric, Chicago, IL, USA). Protein expression levels were quantified via densitometry using ImageJ software, normalized to GAPDH.

**Quantitative Real-time PCR**

Total RNA was isolated from liver using RNAsimple total RNA kits (DP419, TianGen Biotech, Beijing, China) and subsequently reverse-transcribed into cDNA by utilizing the FastQuant RT Kit (KR106, TianGen Biotech, Beijing, China). Real-time PCR amplification was utilized TransStart Green qPCR SuperMix (FP205, TransGen Biotech, Beijing, China) on a LightCycler 96 Quantitative PCR System (Roche, Basel, Switzerland). Relative mRNA expression levels were quantified applying the delta-delta Ct method, normalized to the endogenous reference gene, GAPDH. Primer sequences utilized in this study are detailed in Supplemental Table S1.

**Quantification and Statistical Analysis**

The primary outcome measure of this study was prespecified as the severity of hepatic fibrosis, quantified by the intrahepatic expression of α-SMA and collagen deposition area. Secondary outcomes included longitudinal body weight, serum transaminases (ALT/AST), lipid profiles (CHO, TG, HDL-c, LDL-c), and intrahepatic lactate metabolic markers (LDHA, LDHB, MCT1, PC, PEPCK).

Numerical data were analyzed using GraphPad Prism software (Version 9.0, San Diego, CA, USA) and are presented as the mean ± standard deviation (SD). The 16-week longitudinal body weight data have been analyzed using a two-way repeated-measures ANOVA. Other data were subjected to Shapiro–Wilk and Brown–Forsythe tests for normal distribution and homogeneity of variances. Variables exhibiting normal distribution and homoscedasticity were analyzed using ordinary one-way ANOVA followed by Tukey’s post hoc test. Otherwise, the non-parametric Kruskal–Wallis test was adaptively implemented. A *p* value < 0.05 was considered statistically significant.

## 3. Results

### 3.1. HIIT Improved HFD-Induced MASLD and Hepatic Metabolism in Mice

**Figure 1:**
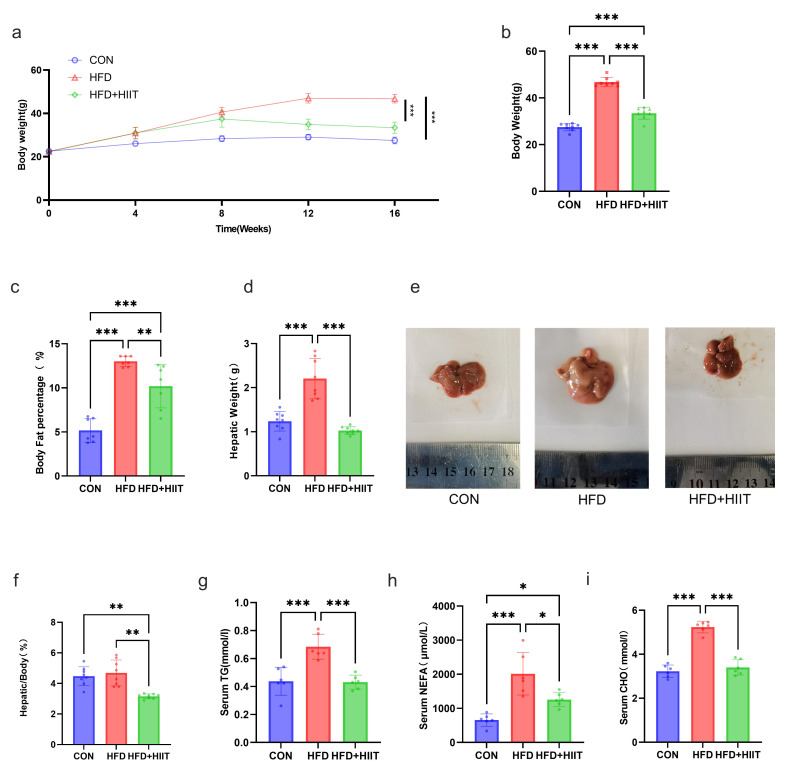
HIIT improved HFD-induced MASLD and hepatic metabolism in mice. Body weights of mice over 16 weeks (**a**) and the body weight (**b**), body fat percentage (**c**), hepatic weight (**d**), liver photograph (**e**), and liver index (liver weight/body weight ratio, *n* = 8) (**f**) at the end of week 16. plasma total triglycerides (TG, **g**), non-esterified fatty acid (NEFA, **h**), and cholesterol (CHO, **i**) levels (*n* = 6). Data are expressed as mean ± standard deviation (SD); * *p* < 0.05, ** *p* < 0.01, and *** *p* < 0.001.

To systematically evaluate the therapeutic efficacy of HIIT on MASLD and hepatic metabolic homeostasis, we employed an HFD-induced murine model. Over the 16-week experimental period (Figure 1a), HFD-fed mice exhibited phenotypic alterations, including markedly elevated body weight (Figure 1b), body fat percentage (Figure 1c), and liver weight (Figure 1d). In terms of appearance, the normal dark-red livers transitioned to a pale, khaki-yellow appearance in the MASLD group (Figure 1e). Strikingly, the 8-week HIIT intervention substantially attenuated body weight gain and body fat accumulation (Figure 1a–c), while completely reversing the gross morphological changes and normalizing both absolute liver weight and the hepatosomatic index (Figure 1d–f). Furthermore, HIIT reduced TG, NEFA, and CHO (Figure 1g–i). Collectively, these data unequivocally demonstrate that HIIT effectively counteracts HFD-induced obesity and systemic metabolic derangements.

### 3.2. HIIT Mitigates Hepatic Lipid Deposition, Oxidative Stress, and Hepatocellular Injury

**Figure 2:**
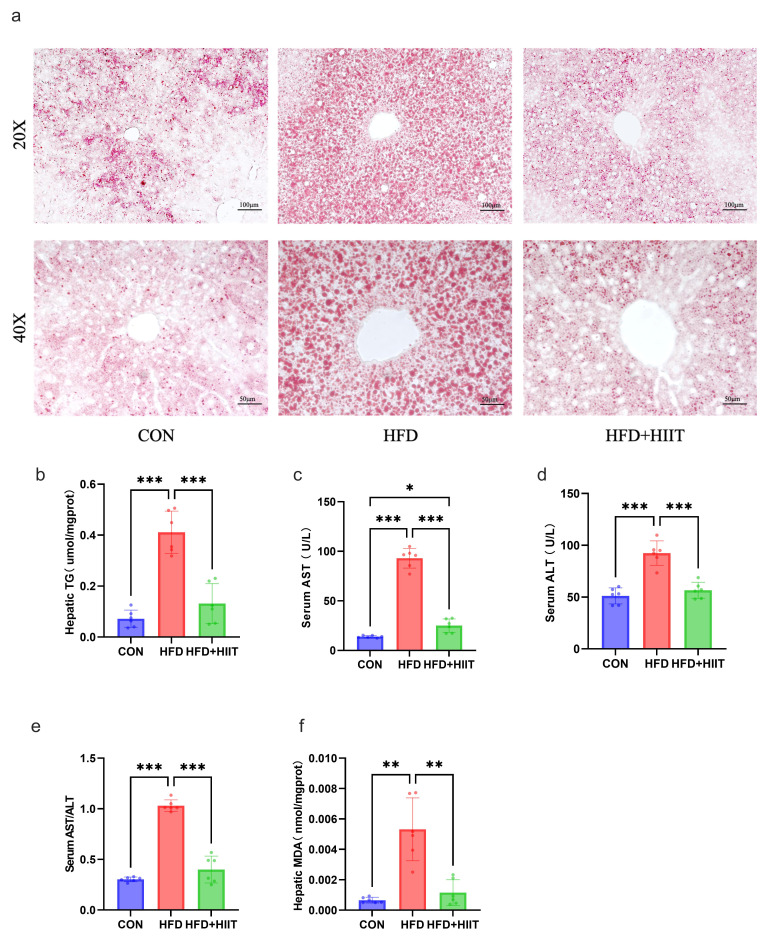
HIIT alleviated hepatic steatosis and reduced liver damage. (**a**) Oil Red O staining of mouse livers (magnification ×20 and ×40). (**b**) TG levels in the livers. Serum levels of ALT (**c**) and AST (**d**) and the ratio of AST to ALT (**e**) in the three groups. (**f**) MDA levels in the liver. Data are presented as mean ± SD (*n* = 6); * *p* < 0.05, ** *p* < 0.01, and *** *p* < 0.001.

Oil Red O staining demonstrated significant lipid droplet accumulation in the liver tissues of HFD mice, while HIIT effectively reduced hepatic lipid droplet content (Figure 2a). Consistent with Oil Red O staining, HFD-induced elevation in hepatic TG levels was significantly suppressed by HIIT (Figure 2b). We also evaluated the impact of HIIT on HFD-induced hepatocellular injury and oxidative stress. Mice in the HFD group exhibited a significant increase in serum concentrations of AST and ALT, as well as AST/ALT ratio (Figure 2c–e). Concurrently, hepatic MDA concentrations were significantly elevated (Figure 2f), indicating HFD-induced lipid peroxidation and oxidative damage in the mice. Notably, HIIT significantly reduced serum AST (Figure 2c), ALT (Figure 2d) and AST/ALT ratio (Figure 2e), and suppressed hepatic MDA accumulation (Figure 2f). Taken together, these findings demonstrate that HIIT provides liver protection against HFD-induced lipotoxicity and oxidative injury.

### 3.3. HIIT Attenuates Hepatic Stellate Cell Activation and MASLD-Associated Liver Fibrosis

**Figure 3:**
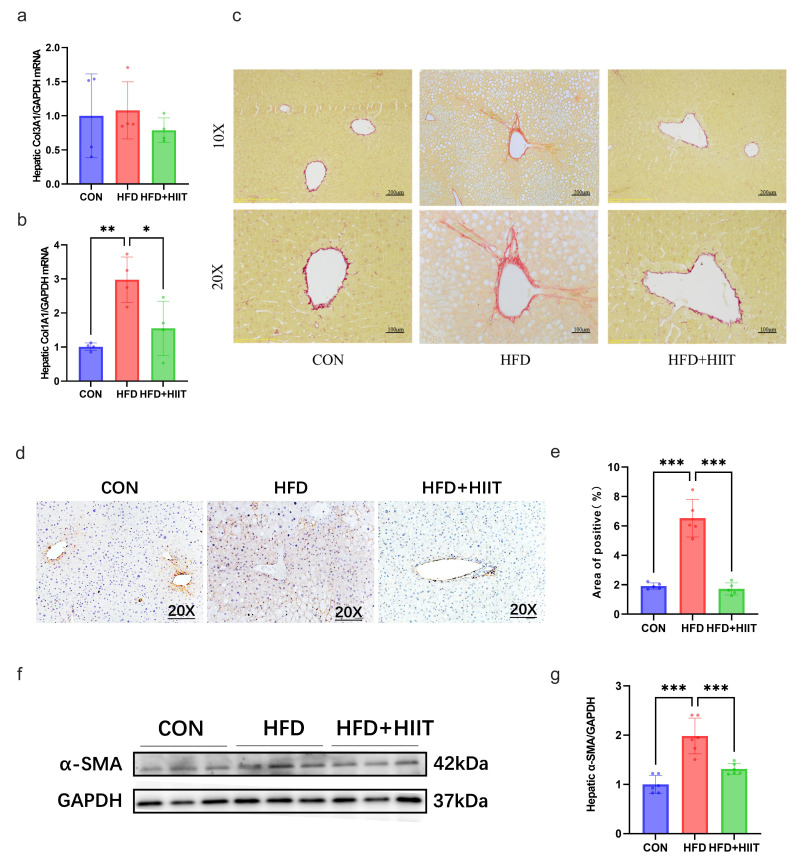
HIIT ameliorated the fibrotic effects in the livers of HFD mice. (**a**,**b**) mRNA expression of collagen type III alpha 1 chain (Col3a1) and collagen type I alpha 1 chain (Col1a1) in the livers of mice (*n* = 4). (**c**) Hepatic Sirius Red staining of mice. (**d**) Immunohistochemical images of alpha-smooth muscle actin (α-SMA) in liver tissue. (**e**) Quantitative analysis of α-SMA (*n* = 5). (**f**,**g**) Protein expression of α-SMA in mouse livers (*n* = 6). Data are expressed as mean ± SD; * *p* < 0.05, ** *p* < 0.01, and *** *p* < 0.001.

Given that unabated lipotoxicity invariably precipitates progressive hepatic fibrogenesis via the activation of hepatic stellate cells (HSCs), we next interrogated the capacity of HIIT to halt this deleterious structural remodeling. While the hepatic mRNA expression of collagen type III alpha 1 chain (Col3a1) remained relatively unaltered across the groups (Figure 3a), collagen type I alpha 1 chain (Col1a1) mRNA was pathologically upregulated by the HFD and significantly blunted following HIIT treatment (Figure 3b). Sirius red staining demonstrated that HFD mice exhibited more severe collagen deposition and fibrosis in liver. Strikingly, the 8-week HIIT intervention markedly restricted this fibrotic expansion (Figure 3c). Furthermore, we assessed the expression of α-SMA, the marker gene of activated HSCs. Immunohistochemical staining revealed a widespread accumulation of α-SMA (indicated by strong positive brown signals) in the MASLD livers; conversely, this signal was substantially diminished in HIIT mice (Figure 3d,e). The results of mRNA and protein expression revealed that HFD markedly elevated α-SMA expression, whereas HIIT statistically downregulated α-SMA expression in the livers of HFD mice (Figure 3f,g). Collectively, these multifaceted data provide compelling evidence that chronic HIIT effectively suppresses HSC activation and limits fibrotic progression in MASLD.

### 3.4. HIIT Ameliorated Hepatic Lactate Metabolism Disorders in MASLD Mice

**Figure 4:**
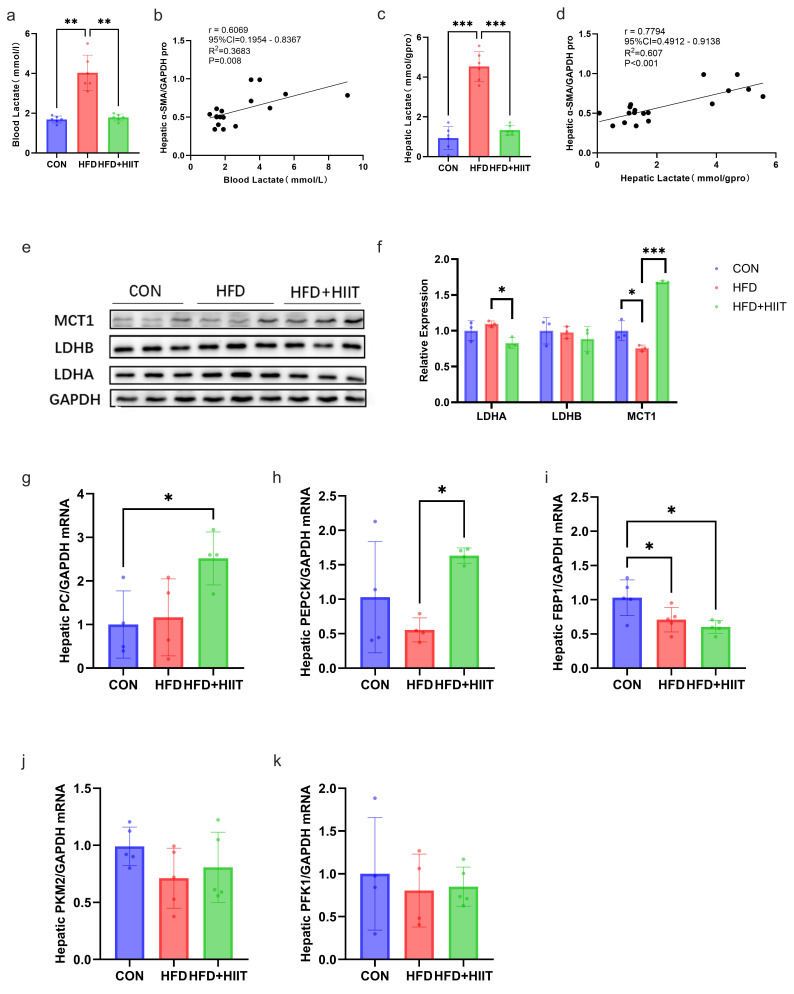
HIIT ameliorated hepatic lactate metabolism disorders in MASLD mice. (**a**,**b**) Serum lactate levels and their correlation with α-SMA levels in the liver (*n* = 6). (**c**,**d**) Hepatic lactate levels and correlation of hepatic lactate with α-SMA levels in the liver (*n* = 6). (**e**,**f**) Protein expression and statistical analysis of monocarboxylate transporter 1 (MCT1), lactate dehydrogenase A (LDHA), and lactate dehydrogenase B (LDHB) in mouse livers (*n* = 3). (**g**–**k**) Relative mRNA expression of gluconeogenic genes pyruvate carboxylase (PC, **g**), phosphoenolpyruvate carboxykinase (PEPCK, **h**), and fructose-bisphosphatase 1 (FBP1, **i**), and glycolytic genes pyruvate kinase M2 (PKM2, **j**) and phosphofructokinase 1 (PFK1, **k**) (*n* = 4–5). Data are presented as mean ± SD; * *p* < 0.05, ** *p* < 0.01, and *** *p* < 0.001.

We next investigated whether the reduced fibrosis in HIIT-treated mice was associated with improvements in hepatic lactate homeostasis. HFD mice showed significant lactate accumulation in both systemic circulation and the liver (Figure 4a,c). This increase was reversed by HIIT, which brought lactate levels back to near-basal values. Linear regression analysis revealed a significant positive correlation between systemic blood lactate levels and hepatic α-SMA expression (Figure 4b; r = 0.6069, 95% CI: 0.1954–0.8367, R^2^ = 0.3683, *p* = 0.008, *n* = 18). More importantly, a highly robust and positive cross-sectional correlation was identified between intrahepatic localized lactate accumulation and α-SMA levels (Figure 4d; r = 0.7794, 95% CI: 0.4912–0.9138, R^2^ = 0.607, *p* < 0.001, *n* = 18). Taken together, our data demonstrates a strong correlation between intrahepatic lactate clearance and the suppression of hepatic stellate cell activation in MASLD. While this cross-sectional association does not imply direct causality, the tight coupling between these two pathways suggests that lactate homeostasis is a critical metabolic component of the therapeutic adaptations induced by HIIT.

We then analyzed the enzymes and transporters responsible for lactate balance. LDHA is an important enzyme in glycolysis that converts pyruvate to lactate, whereas LDHB facilitates the reverse reaction. HIIT reduced the protein expression of LDHA, which catalyzes lactate production, but had no effect on LDHB (Figure 4e,f). At the same time, the expression of MCT1, a transporter for lactate efflux, was lower in HFD mice but significantly increased after HIIT intervention (Figure 4e,f). These data demonstrate that HIIT can promote the efflux and transport of hepatic lactate, which effectively reduces lactate accumulation in the liver in MASLD.

Since lactate is a substrate for gluconeogenesis, we also checked the expression of key metabolic enzymes. HIIT increased the mRNA levels of the early gluconeogenic enzymes phosphoenolpyruvate carboxykinase (PEPCK, Figure 4g,h). However, the expression of the downstream enzyme fructose-bisphosphatase 1 (FBP1) was decreased in HFD+HIIT group (Figure 4j). The glycolytic enzymes pyruvate kinase M2 (PKM2) and phosphofructokinase 1 (PFK1) did not change significantly among the groups (Figure 4i,k). Together, these results show that HIIT reduces lactate levels by decreasing production through LDHA and increasing efflux via MCT1, potentially shifting the metabolic flux toward gluconeogenesis.

## 4. Discussion

Our study demonstrates that HIIT intervention effectively mitigates HFD-induced MASLD and hepatic fibrosis by remodeling the hepatic lactate metabolism. HIIT suppressed intrahepatic lactate accumulation by downregulating lactate production and upregulating lactate transporters and clearance-related genes. These findings suggest that the therapeutic effects of HIIT in MASLD are intricately linked to the restoration of hepatic lactate homeostasis.

Previous research indicates that MASLD is fundamentally driven by chronic energy imbalance and ectopic lipid deposition [15,16]. Chronic HFD feeding leads to an MASLD phenotype, involving weight gain, liver steatosis, and increased serum LDL and ALT levels [17]. In our study, HFD-fed model recapitulated this phenotype, as evidenced by significant increases in the hepatosomatic index and intrahepatic triglyceride content. Beyond lipid accumulation, the observed elevations in serum transaminases (ALT/AST) and hepatic MDA concentrations indicate severe hepatocellular injury and oxidative stress. While exercise is increasingly recognized as an effective treatment for MASLD [18,19], HIIT has demonstrated superior efficacy in enhancing hepatic insulin sensitivity and suppressing lipid synthesis compared to conventional aerobic modalities [20]. These metabolic benefits are likely mediated by the acute physiological adaptations to HIIT—including rapid glucose mobilization and enhanced insulin secretion—which collectively suppress lipid synthesis. Consistent with these mechanisms, our study found 8-week HIIT intervention effectively counteracted the HDF-induced metabolic derangement, restored liver morphology and function.

The progression of MASLD frequently culminates in hepatic fibrosis, which is closely associated with hepatocellular stress, chronic inflammation, and the fibrotic process [21,22]. This process is driven by the activation of hepatic stellate cells (HSCs), which transdifferentiate from a non-proliferative, lipid-storing cell state into proliferative, myofibroblast-like cells [23]. Activated HSCs are responsible for the excessive accumulation of α-SMA, type I collagen, and ECM proteins in the liver [24,25]. Exercise training reduces the inflammatory response and downregulates the expression of fibrotic marker genes in a murine model of liver fibrosis [26,27]. Xavier et al. demonstrated that a 16-week HIIT intervention in mice with HFD-induced MASH and fibrosis significantly reduced the area positively stained for α-SMA via immunohistochemistry which indicated that HIIT markedly attenuated HSC activation and ameliorated the progression of liver fibrosis [28]. Consistent with these findings, our study provides additional evidence to support this conclusion. Both Sirius Red staining and α-SMA protein analysis confirmed that an 8-week HIIT regimen markedly reduced collagen deposition and HSC activation.

The liver serves as the primary organ for systemic lactate homeostasis, coordinating its clearance through oxidation or resynthesized into glucose via gluconeogenesis [29,30,31]. However, hepatic metabolic processes may become impaired in chronic liver diseases, leading to lactate accumulation [32]. Emerging evidence indicates that abnormal lactate accumulation directly drives MASLD pathogenesis through novel protein lactylation mechanisms, where histone and non-histone modifications cooperate to accelerate the progression of hepatic injury [33,34]. Consistent with these reports, our study observed elevated hepatic and circulating lactate levels in MASLD mice, which was accompanied by a marked upregulation of LDHA. Crucially, the strong positive correlation between intrahepatic lactate abundance and α-SMA protein levels suggests that this abnormal lactate pool is not merely a metabolic byproduct, but a prominent metabolic factor tightly coupled with HSC activation and the ensuing fibrotic expansion.

Exercise is known to improve MASLD through multiple mechanisms, such as the mitigation of oxidative stress [35], suppression of inflammatory responses [36], enhancement of lipophagy [37], and promotion of hepatic autophagy [38]. Unlike conventional exercise, HIIT improves hepatic steatosis by a refined coordination between lactate production and clearance, which in turn promotes fatty acid oxidation and energy metabolism [39,40,41]. Our findings reveal that HIIT significantly downregulated both the protein and mRNA expression of *LDHA*, directly reducing hepatic lactate production. Concurrently, the marked upregulation of key gluconeogenic enzymes suggests that HIIT enhances the liver’s function to sequester and utilize accumulated lactate. The unchanged expression of key glycolytic enzymes—such as PKM2 and PFK1—indicates that HIIT selectively shunts lactate toward gluconeogenic flux rather than anaerobic glycolysis.

Several limitations of the current study warrant mention. Although young male murine cohorts were utilized to minimize hormone-driven metabolic variance, this homogeneous cohort cannot fully recapitulate the clinical heterogeneity of age-dependent MASLD. In addition, the absence of a normal diet plus HIIT control group precludes determining whether HIIT’s benefits are disease-specific or reflect generalized exercise adaptations. Moreover, our study cannot unequivocally demonstrate a direct causal relationship between lactate reduction and hepatic fibrosis, necessitating further validation through in vitro cellular assays in future investigations. Lastly, because this initial phase was strictly focused on localized hepatic lactate turnover and its coupling with fibrotic remodeling, systemic fasting glucose and insulin profiles were not characterized. Despite these inherent limitations, our work nonetheless provides valuable mechanistic insights into the therapeutic efficacy of HIIT against hepatic metabolic derangement and fibrogenesis.

## 5. Conclusions

Our study demonstrates that an HIIT intervention can alleviate the symptoms of MASLD and hepatic fibrosis by remodeling the hepatic lactate metabolic axis, with this effect being mediated by the regulation of lactate turnover. We also found a close association between the restoration of hepatic lactate homeostasis and the suppression of hepatic stellate cell activation. Additionally, based on the Western blotting and mRNA results, we believe that HIIT regulates lactate metabolism by inhibiting its production via LDHA downregulation while promoting its efflux and gluconeogenic shunting through MCT1 and gluconeogenic enzymes. Therefore, HIIT might be a promising non-pharmacological therapeutic strategy for treating MASLD-associated hepatic fibrosis.

## Data Availability

The raw data supporting the conclusions of this article will be made available by the authors on request.

## Supplementary Materials

The following supporting information can be downloaded at https://www.mdpi.com/article/10.3390/metabo16060413/s1. Table S1: List of primers used for the PCR analysis.

## Abbreviations

The following abbreviations are used in this manuscript:

ALT	Alanine aminotransferase
AST	Aspartate aminotransferase
BAT	Brown adipose tissue
CHO/TC	Total cholesterol
Col1a1	Collagen type I alpha 1 chain
Col3a1	Collagen type III alpha 1 chain
CON	Control group
eWAT	Epididymal white adipose tissue
FBP1	Fructose-bisphosphatase 1
GADPH	Glyceraldehyde-3-phosphate dehydrogenase
HDL-c	High-density lipoprotein cholesterol
HFD	High-fat diet
HIIT	High-intensity interval training
HSCs	Hepatic stellate cells
IHC	Immunohistochemistry
iWAT	Inguinal white adipose tissue
LDHA	Lactate dehydrogenase A
LDHB	Lactate dehydrogenase B
LDL-c	Low-density lipoprotein cholesterol
MASLD	Metabolic dysfunction-associated steatotic liver disease
MCT1	Monocarboxylate transporter 1
MDA	Malondialdehyde
NEFA	Non-esterified fatty acids
PC	Pyruvate carboxylase
PEPCK	Phosphoenolpyruvate carboxykinase
PFK1	Phosphofructokinase 1
PKM2	Pyruvate kinase M2
PVDF	Polyvinylidene difluoride
qPCR	Quantitative real-time polymerase chain reaction
SD	Standard deviation
TG	Triglycerides
α-SMA	Alpha-smooth muscle actin
