# Supplementary material for: High-Intensity Interval Training Attenuates Hepatic Fibrosis by Remodeling Lactate Metabolism in MASLD
Source: Metabolites. 2026 Jun 13;16(6):413. doi: 10.3390/metabo16060413 (PMC13303855; doi:10.3390/metabo16060413)
Supplement: Supplementary file 1 [file metabolites-16-00413-s001.zip › metabolites-4341082-supplementary.pdf]

Supplemental Table S1

List of primers used for the PCR analysis.

Gene	Sequence （5’-3’）
GAPDH	Forward5’-ACAGCAACAGGGTGGTGGAC -3’
	Reverse5’-TTT GAG GGT GCAGCGAACTT -3’
LDHA	Forward5’-TGTGGCAGACTTGGCTGAGA-3’
	Reverse5’-CTGAGGAAGACATCCTCATTGATTC -3’
LDHB	Forward5’-CCTCAGATCGTCAAGTACAGTCC-3’
	Reverse5’-ATCACGCGGTGTTTGGGTAAT -3’
COL1A1	Forward5’-ACATGTTCAGCTTTGTGGACC -3’
	Reverse5’-TAGGCCATTGTGTATGCAGC -3’
COL3A1	Forward5’-CGAGGTAACAGAGGTGAAAGA -3’
	Reverse5’-AACCCAGTATTCTCCGCTCTT -3’
PFK1	Forward5’-CCATCAGCAACAATGTGCCTGG -3’
	Reverse5’-GATGCCATCGCTCACTTCTAGG -3’
PKM2	Forward5’-ACTCGGGCTGAAGGCAGTGA -3’
	Reverse5’-TGTGGGGTCGCTGGTAATGG -3’
PC	Forward5’-GCCCAGAAGTTGCTACATTACCT -3’
	Reverse5’-CTCACATTGACAGGGATTGGA -3’
PEPCK	Forward5’-CACCATCACCTCCTGGAAGA -3’
	Reverse5’-GGGTGCAGAATCTCGAGTTG -3’
PDHA1	Forward5’-GTGAGAACAACCGCTATGGCATG-3’
	Reverse5’-CGCAAACTTTGTTGCCTCTCGG-3’
CS	Forward5’-GGACAATTTTCCAACCAATCTGC-3’
	Reverse5’-TCGGTTCATTCCCTCTGCATA-3’
